# Profile of Cerebrospinal microRNAs in Fibromyalgia

**DOI:** 10.1371/journal.pone.0078762

**Published:** 2013-10-25

**Authors:** Jan L. Bjersing, Christopher Lundborg, Maria I. Bokarewa, Kaisa Mannerkorpi

**Affiliations:** 1 Department of Rheumatology and Inflammation Research, Institute of Medicine, Sahlgrenska Academy, University of Gothenburg, Göteborg, Sweden; 2 Department of Anaesthesiology and Intensive Care Medicine, Institute of Clinical Sciences, The Sahlgrenska Academy, University of Gothenburg, Göteborg, Sweden; 3 University of Gothenburg Centre for Person-centred Care (GPCC), Sahlgrenska Academy, University of Gothenburg, Göteborg, Sweden; The James Cook University Hospital, United Kingdom

## Abstract

**Introduction:**

Fibromyalgia (FM) is characterized by chronic pain and reduced pain threshold. The pathophysiology involves disturbed neuroendocrine function, including impaired function of the growth hormone/insulin-like growth factor-1 axis. Recently, microRNAs have been shown to be important regulatory factors in a number of diseases.

The aim of this study was to try to identify cerebrospinal microRNAs with expression specific for FM and to determine their correlation to pain and fatigue.

**Methods:**

The genome-wide profile of microRNAs in cerebrospinal fluid was assessed in ten women with FM and eight healthy controls using real-time quantitative PCR. Pain thresholds were examined by algometry. Levels of pain (FIQ pain) were rated on a 0-100 mm scale (fibromyalgia impact questionnaire, FIQ). Levels of fatigue (FIQ fatigue) were rated on a 0-100 mm scale using FIQ and by multidimensional fatigue inventory (MFI-20) general fatigue (MFIGF).

**Results:**

Expression levels of nine microRNAs were significantly lower in patients with FM patients compared to healthy controls. The microRNAs identified were miR-21-5p, miR-145-5p, miR-29a-3p, miR-99b-5p, miR-125b-5p, miR-23a-3p, 23b-3p, miR-195-5p, miR-223-3p.

The identified microRNAs with significantly lower expression in FM were assessed with regard to pain and fatigue. miR-145-5p correlated positively with FIQ pain (r=0.709, p=0.022, n=10) and with FIQ fatigue (r=0.687, p=0.028, n=10).

**Conclusion:**

To our knowledge, this is the first study to show a disease-specific pattern of cerebrospinal microRNAs in FM.

We have identified nine microRNAs in cerebrospinal fluid that differed between FM patients and healthy controls. One of the identified microRNAs, miR-145 was associated with the cardinal symptoms of FM, pain and fatigue.

## Introduction

Fibromyalgia (FM) is a disorder characterized by chronic pain and reduced pain threshold[1]. The pathophysiology of FM involves disturbed neuroendocrine function, including impaired function of the growth hormone/insulin-like growth factor-1 axis[2-4]. FM is also associated with pronounced fatigue[5-7]. FM is regarded as a multifactorial disorder, possibly involving both environmental and genetic factors. 

Clustering of FM in families is common[8,9] and a number of candidate FM genes have been proposed. So far, most of the genetic studies have been fairly small and findings have been partly divergent [10]. However, a large scale candidate study was recently performed that identified three novel candidate genes[11] associated with the disorder. 

Since FM is a chronic condition, it is likely that the ongoing disease process and environmental influences leads to long-term changes in gene expression. Evidence is emerging for the involvement of such epigenetic processes in chronic pain[12] but has not been studied specifically in patients with FM. 

MicroRNAs have in recent years been identified as important modulators of gene expression in disease processes and physiological pathways. The microRNAs are highly evolutionary conserved, short non-coding RNA molecules, approximately 20-22 nucleotides in length. They inhibit gene expression post-transcriptionally either by inhibition of translation or by degradation of the target messenger RNA. MicroRNAs are believed to regulate at least 30% of human genes[13] and individual microRNAs can repress hundreds of genes[14]. Conversely, one messenger RNA can be the target of several microRNAs. These regulatory networks can control complex programs of gene expression[15] and allows precise adjustments of protein output[14]. Changes in a single or small group of microRNAs may therefore sensitively reflect changes involving a large number of different messenger RNAs. 

MicroRNAs are important in the regulation of many processes in the central nervous system (CNS). They have been implicated in several disease states in the CNS[16] and may play important roles in the regulation of neuronal plasticity under stress[17]. 

To our knowledge expression of cerebrospinal microRNAs in FM has not been previously studied. 

The aim of this study was to try to identify cerebrospinal microRNAs with expression specific for FM and also to determine their relation to the cardinal FM symptoms of fatigue and pain.

## Materials and Methods

### Study design and subjects

Ten patients with FM were compared to eight age-matched healthy controls. Cerebrospinal fluid (CSF) was collected at rest by lumbar puncture through the L3/L4 interspace. Serum was also collected at the same time from eight of the ten FM patients. Collected CSF and serum samples were centrifuged at 800 g for 3 minutes, aliquoted, and stored frozen at -70°C until use. Samples of FM patients were taken during participation in a regular outdoors exercise program, of low to moderate levels, as reported earlier [18]. Patients did not participate in exercise on the day the sample was collected. For characteristics of patients and healthy controls, see Table 1. 

**Table 1:** Characteristics and pharmacologic treatment of FM patients and healthy controls.

	**Healthy controls (n=8)**	**FM patients (n=10)**
Age, years	58.5 (38.0 to 68.3)	48.5 (37.8 to 53.0)
Symptom duration, years	-	9 (6-16)
Tender points, n	-	15 (13-16)
Pain threshold	-	198.3 (168.0 to 220.6)
FIQ pain	-	70.3 (53.4 to 83.9)
FIQ fatigue	-	89 (66.5 to 92.0)
MFIGF	-	18.5 (13.6 to 20.0)
Antidepressants, yes	0 (0%)	6 (60%)
Analgesics, yes	2(25%)	9 (90%)
BMI, kg/m^2^	26.5 (22.5 to 28.7)	28.4 (25.8 to 29.7)

Median values and interquartile range are indicated

Healthy controls who had no current pain or history of long-term pain were recruited among patients receiving spinal anaesthesia for gynaecological or urological interventions (n=8). Patients not speaking or reading Swedish, or having any severe somatic or psychiatric disease were excluded.

FM patients were recruited by advertisements in local newspapers. Criteria for inclusion of FM patients: Women with FM, aged 20 to 60 years, and with interest in exercising outdoors twice a week for 15 weeks, willing to participate at blood tests[18]. FM was defined by the ACR 1990 criteria[1]: a history of long-lasting generalized pain and with pain in at least 11 of 18 tender points examined by manual palpation. Criteria for exclusion of FM patients: Patients not speaking or reading Swedish. Having any severe somatic or psychiatric disease. The participation of these FM patients in an outdoors exercise program indicates reasonably good somatic health.

The median age of FM patients was 48.5 (37.8 to 53.0) years and of healthy controls 58.5 (38.0 to 68.3, n.s.) years. The symptom duration of FM patients was 9 (6-16) years and the average number of tender points was 15 (13-16). Ninety percent of FM patients were taking analgesics during the study and 60% were taking antidepressants or sedatives. The median body mass index (BMI) tended to be higher in FM patients 28.4 kg/m^2^ (25.8 to 29.7) compared to healthy controls 26.5 (22.5 to 28.7 n.s).

### Clinical measurements

The pain threshold was examined by using an algometer (Somedic Production AB, Sollentuna, Sweden), measured in kilopascals (kPa)[19]. The pain threshold was measured in two tender point locations in the upper and lower extremities, respectively. The mean value was applied, and a higher value indicates better health. Levels of pain (FIQ pain) were rated on a visual analogue scale (0-100 mm, Fibromyalgia Impact Questionnaire, FIQ)[20]. Levels of fatigue (FIQ fatigue) were also rated on a visual analogue scale (0-100 mm) using FIQ which gives an estimation of global fatigue, as well as with the Multidimensional Fatigue Inventory (MFI-20)[21] subscale of General Fatigue (MFIGF, range 4-20), which estimates fatigue by questions related to feeling “fit”, “tired” and “rested” . Both instruments reflect fatigue during the last week, and a higher score indicates more severe fatigue.

### Sample preparation of circulating microRNA

Isolation of total RNA from CSF was conducted at Exiqon Services, Denmark. Total RNA was extracted from serum using the Qiagen miRNeasy Mini Kit. CSF was thawed on ice and centrifuged at 3000 x g for 5 min in a 4°C microcentrifuge. An aliquot of 200 μL of CSF per sample was transferred to a new microcentrifuge tube and 750 μl of a Qiazol mixture containing 1.25 μg/mL of MS2 bacteriophage RNA was added to the CSF. The tube was mixed and incubated for 5 min followed by the addition of 200 μL chloroform. The tube was mixed, incubated for 2 min and centrifuged at 12,000 x g for 15 min in a 4°C microcentrifuge. The upper aqueous phase was transferred to a new microcentrifuge tube and 1.5 volume of 100% ethanol was added. The contents were mixed thoroughly and 750 μL of the sample was transferred to a Qiagen RNeasy Mini spin column in a collection tube followed by centrifugation at 15,000 x g for 30 sec at room temperature. The process was repeated until all remaining sample had been loaded. The Qiagen RNeasy Mini spin column was rinsed with 700 μL Qiagen RWT buffer and centrifuged at 15,000 x g for 1 min at room temperature followed by another rinse with 500 μL Qiagen RPE buffer and centrifuged at 15,000 x g for 1 min at room temperature. A rinse step (500 μL Qiagen RPE buffer) was repeated twice. The Qiagen RNeasy Mini spin column was transferred to a new collection tube and centrifuged at 15,000 x g for 2 min at room temperature. The Qiagen RNeasy Mini spin column was transferred to a new microcentrifuge tube and the lid was left uncapped for 1 min to allow the column to dry. Total RNA was eluted by adding 50 μL of RNase-free water to the membrane of the Qiagen RNeasy mini spin column and incubating for 1 min before centrifugation at 15,000 x g for 1 min at room temperature. The RNA was stored in a -80°C freezer. 

### microRNA real-time qPCR

Amplification and detection of microRNAs was conducted at Exiqon Services, Denmark. RNA was reverse transcribed in 40μl reactions using the miRCURY LNA Universal RT microRNA PCR, Polyadenylation and cDNA synthesis kit (Exiqon). An artificial RNA (RNA spike-in) was added to the reverse transcription step. This control was used to confirm that the reverse transcription and amplification occurred with equal efficiency in all samples. cDNA was diluted and assayed in PCR reactions according to the protocol for miRCURY LNA Universal RT microRNA PCR; each microRNA was assayed by qPCR on the microRNA Ready-to-Use PCR microchip (Exiqon, Denmark, Cat No 203608) for detection of human microRNAs. Negative controls excluding template from the reverse transcription reaction were included and profiled like the samples. The amplification was performed in a LightCycler 480 Real-Time PCR System (Roche) in 384 well plates. The amplification curves were analyzed using the Roche LC software, both for determination of crossing point (Cp) value, by the 2nd derivative method, and for melting curve analysis.

### Data analysis

A total of 742 human microRNAs and three snRNA reference genes were assayed in each sample. The three small nuclear RNA (snRNA) reference genes were not included in the subsequent analysis. MicroRNAs with a Cp<40 in the negative control were excluded from analysis. A detection threshold of Cp<38 was defined to achieve optimal information quality[22]. MicroRNAs with no detectable expression in any samples were omitted from further analysis.

Sixty microRNAs were present in 50% or more of subjects reflecting low levels of microRNAs detectable in the CSF and indicating a CSF-specific pattern of microRNA expression. Serum samples were available for eight of ten FM patients. Therefore, it was possible to determine the presence or absence in serum of the nine microRNAs that differed in CSF between FM and healthy controls. All these nine microRNAs were also present in the serum of these eight FM patients.

### Normalisation

Normalization of the microRNA RT-qPCR data was performed with rank normalisation by calculation of fractional rank [23]. First, the Cp value of each individual microRNA species was ranked within each sample. The highest amount of microRNA in one sample was assigned the highest rank in that sample. In the next step, the rank of one microRNA was divided by the total number of detectable microRNAs in the sample. This was the fractional rank. 

Thus, for each microRNA species in a sample the fractional rank was calculated using the following formula: *Fractional rank = individual rank* (*rank of one individual microRNA species in the sample*)*/*(*number of all expressed microRNAs in sample +1*)

### Statistical analysis

Fractional ranks of FM patients and healthy controls were compared using with Mann-Whitney U Test. Group comparisons between healthy controls and FM patients were performed for microRNAS expressed with a Cp<38 in 50% or more of subjects. Thus, sixty microRNAs were compared. Bonferroni was used to define the critical p-value for multiple comparisons. The critical p-value is 0.0008333 for 60 comparisons and nine microRNAs were significantly different between FM patients and healthy controls (table 2). Subsequently, relations between these nine identified microRNAs and typical FM symptoms were examined with the Spearman correlation coefficient. Statistical analysis were performed using SPSS (version 19.0.0 for Mac). 

**Table 2:** List of microRNAs with significantly different expression between FM patients and healthy controls.

	**Healthy controls**	**FM**	**Comparison of groups**
	**Fractional rank**	**Fractional rank**	**p-value^a^**
miR-21-5p	0.974 (0.964 to 0.977)	0.715 (0.306 to 0.804)	4.6x10^-5^
miR-145-5p	0.956 (0.880 to 0.966)	0.080 (0.026 to 0.262)	4.6x10^-5^
miR-29a-3p	0.240 (0.133 to 0.563)	0.021 (0.017 to 0.037)	9.1x10^-5^
miR-99b-5p	0.636 (0.476 to 0.725)	0.164 (0.015 to 0.359)	1.8x10^-4^
miR-125b-5p	0.981 (0.971 to 0.988)	0.912 (0.900 to 0.947)	3.2x10^-4^
miR-23a-3p	0.937 (0.883 to 0.956)	0.362 (0.215 to 0.654)	3.2x10^-4^
miR-23b-3p	0.693 (0.581 to 0.772)	0.223 (0.017 to 0.329)	5.5x10^-4^
miR-195-5p	0.703 (0.344 to 0.773)	0.025 (0.019 to 0.128)	5.5x10^-4^
miR-223-3p	0.715 (0.560 to 0.885)	0.054 (0.020 to 0.303)	5.5x10^-4^

MicroRNAs are listed in order of significance, starting with the lowest p-values. Normalized levels of microRNAs are expressed as the fractional rank. Higher fractional rank represents higher levels of microRNA. Median fractional rank values and interquartile range are indicated. ^a^ Mann-Whitney U test.

### Ethics

*The study was approved by the ethics committee of Sahlgrenska University Hospital. Written and verbal information was given to all patients, and written consent was obtained from all patients.*

Trial registration: **ClinicalTrials.gov** NCT00643006

## Results

### Differences in cerebrospinal microRNA expression between FM patients and healthy controls

MicroRNA expression was compared between FM patients and healthy controls. Nine microRNAs in CSF were significantly different in expression between patients with FM and healthy controls after applying Bonferroni correction for multiple testing (Table 2 and Table S1). 

Because the controls were older, a separate comparison was made excluding the two oldest patients in the control group. The median age of this subset of healthy controls (n=6) was 47.5 (37.8 to 53) years compared to 48.5 (37.8 to 53.0, n=10, n.s.) years in FM patients. The median BMI of this subset of healthy controls was 27.5 (23.2 to 31.6, n=6) kg/m^2^ compared to 28.4 kg/m^2^ (25.8 to 29.7, n=10, n.s.) in patients with FM. 

The same nine microRNAs that initially were identified as differently expressed (Table 2) were also the nine highest ranking microRNAs (ranked according to p-value) after matching groups with regard to age, by using the reduced subset of six healthy controls. Four of these microRNAs, miR-21-5p, miR-145-5p, miR-223-3p and miR-29a-3p remained statistically significant after applying Bonferroni correction for multiple testing. The remaining five microRNAS, miR-99b-5p (p<0.001), miR-125b-5p (p<0.002), miR-23a-3p (p<0.002), miR-23b-3p (p<0.003) and miR-195-5p (p<0.003) did not reach statistical significance.

### Disease associated microRNAs and symptom severity

The relation of the nine identified microRNAs to pain and fatigue was subsequently tested in the FM patients. The correlations are shown in Table 3. The identified FM-associated microRNAs in cerebrospinal fluid were assessed with regard to pain and fatigue. miR-145-5p was correlated with FIQ pain (r=0.709, p=0.022, n=10) and with FIQ fatigue (r=0.687, p=0.028, n=10). 

**Table 3:** Relation of disease-associated CSF microRNAs with cardinal symptoms in FM.

	**Pain threshold**	**FIQ pain**	**FIQ fatigue**	**MFIGF**
miR-21-5p	r^s^=-0.382	r^s^=0.018	r^s^=-0.103	r^s^=0.138
	p=0.276	p=0.960	p=0.776	p=0.704
	n=10	n=10	n=10	n=10
miR-145-5p	r^s^=-0.115	r^s^=0.709	r^s^=0.687	r^s^=0.276
	p=0.751	**p=0.022**	**p=0.028**	p=0.440
	n=10	n=10	n=10	n=10
miR-29a-3p	r^s^=-0.298	r^s^=-0.055	r^s^=-0.192	r^s^=0.277
	p=0.403	p=0.881	p=0.595	p=0.439
	n=10	n=10	n=10	n=10
miR-99b-5p	r^s^=-0.553	r^s^=-0.237	r^s^=0.052	r^s^=0.145
	p=0.097	p=0.510	p=0.887	p=0.690
	n=10	n=10	n=10	n=10
miR-125b-5p	r^s^=0.103	r^s^=0.201	r^s^=0.174	r^s^=0.258
	p=0.776	p=0.578	p=0.631	p=0.472
	n=10	n=10	n=10	n=10
miR-23a-3p	r^s^=0.018	r^s^=-0.103	r^s^=0.036	r^s^=0.088
	p=0.960	p=0.777	p=0.920	p=0.809
	n=10	n=10	n=10	n=10
miR-23b-3p	r^s^=-0.164	r^s^=-0.285	r^s^=0.055	r^s^=0.32
	p=0.651	p=0.425	p=0.881	p=0.367
	n=10	n=10	n=10	n=10
miR-195-5p	r^s^=-0.370	r^s^=-0.018	r^s^=0.395	r^s^=0.464
	p=0.293	p=0.960	p=0.258	p=0.177
	n=10	n=10	n=10	n=10
miR-223-3p	r^s^=-0.286	r^s^=-0.310	r^s^=0.137	r^s^=0.447
	p=0.424	p=0.383	p=0.705	p=0.196
	n=10	n=10	n=10	n=10

Spearmans rho (r^s^) of miRNA relative expression levels vs. symptom severity levels , two-tailed significance (p , and number of subjects (n) are presented.

## Discussion

The pathophysiology of chronic pain in FM involves disturbed neuroendocrine function with contribution of the growth hormone axis. The pathogenesis of fatigue in FM is less well understood. MicroRNAs are increasingly recognised as important regulatory factors with diagnostic and therapeutic potential in many human diseases. 

In this study we identified several microRNAs that were differently expressed in the CSF of FM patients and the healthy controls. These difference may reflect pathological changes in the central nervous system. Changes in relative microRNA levels may also represent adaptive responses to minimise the effects of the disease and its symptoms. 

miR-21 is involved in glial[24,25] and neuronal[26,27] responses to injury in the central nervous system[28]. Identifying alterations in cerebrospinal microRNA networks in FM may give new leads to the pathogenesis of this condition.

miR-145-5p correlated with pain and fatigue in the FM patients. miR-145 inhibits growth[29] and is a potential modulator of the insulin-like growth factor pathway[30]. miR-145 was downregulated in endometriosis patients[31], in ulcerative colitis patients[32] and in experimental studies of prenatal stress[33]. However, in this small sample of patients with FM, post-hoc analysis did not show any significant correlation between miR-145-5p and serum-free or total insulin-like growth factor 1 (data not shown).

miR-29 is upregulated during aging in mice[34] and its inhibition reduced cellular aging[35]. miR-29 is decreased in Alzheimers disease[36], Parkinsons disease[37] and myotonic dystrophy type 1[38]. Together with miR-125, miR-99, miR-223, and miR-145, miR-29 was upregulated during endometriosis[39]. In an exercise study in animals miR-29 was increased leading to improved cardiac function after exercise training[40].

miR-23b is a regulator of µ-opioid receptor expression and responds to long-term morphine treatment[41]. MiR-23b is repressed in several autoimmune conditions including multiple sclerosis, systemic lupus erythematosus and experimental autoimmune encephalitis[42]. Conversely, miR-23b is anti-inflammatory and its overexpression suppresses autoimmune pathogenesis under experimental conditions[42]. 

miR-195-5p was reduced in FM patients compared to healthy controls. miR195 is involved in energy metabolism[43] and growth[44,45] and appears to suppress BDNF protein levels in the prefrontal cortex[46]. MiR-195 protects against hypoperfusion induced dementia by inhibition of beta-amyloid production[47,48]. 

miR-223 also showed neuroprotective activity by the targeting of glutamate receptors[49] and is changed in the mouse prefrontal cortex after inflammatory pain[50]. 

All the FM-associated microRNAs were significantly reduced compared to healthy controls. Among FM patients, miR-145-5p was correlated with pain and fatigue.

A limitation of this study is the small number of subjects. Therefore, these results are indicative and validation in other groups of patients and with larger study groups would be valuable.

The relative amount of miR-145-5p was strongly downregulated in FM patients. Nevertheless, among FM patients higher levels of pain and fatigue corresponded to higher amounts of miR-145-5p. An explanation is that higher levels of miR-145-5p in FM patients represent an adaptive but ineffective response to pain and fatigue. This hypothesis should be investigated in longitudinal studies with interventions that change pain and fatigue in FM.

## Conclusion

This study shows for the first time a disease-specific pattern of cerebrospinal microRNAs in FM patients. One of the identified microRNAs, miR-145 was associated with the cardinal symptoms of FM, pain and fatigue.

## Supporting Information

Table S1 **Complete list of microRNAs included in the comparative analysis between FM patients and healthy controls.** All microRNAs included in the group comparison are listed in order of significance, starting with the lowest p-values. Normalized levels of microRNAs are expressed as the fractional rank. Higher fractional rank represents higher levels of microRNA. Median fractional rank values and interquartile range are indicated. ^a^Mann-Whitney U test.
(DOC)
