# Supplementary material for: Profile of Cerebrospinal microRNAs in Fibromyalgia
Source: PLoS One. 2013 Oct 25;8(10):e78762. doi: 10.1371/journal.pone.0078762 (PMC3808359; doi:10.1371/journal.pone.0078762)
Supplement: Table S1 — Complete list of microRNAs included in the comparative analysis between FM patients and healthy controls. All microRNAs included in the group comparison are listed in order of significance, starting with the lowest p-values. Normalized levels of microRNAs are expressed as the fractional rank. Higher fractional rank represents higher levels of microRNA. Median fractional rank values and interquartile range are indicated. aMann-Whitney U test. (DOC) [file pone.0078762.s001.doc]

Table S1. Complete list of microRNAs included in the comparative analysis between FM patients and healthy controls.

|  | **Healthy controls** | **FM** | **Comparison of groups** |
| --- | --- | --- | --- |
|  | **Fractional rank** | **Fractional rank** | **p-value^a^** |
| miR-21-5p | 0.974 (0.964 to 0.977) | 0.715 (0.306 to 0.804) | 4.6x10^-5^ |
| miR-145-5p | 0.956 (0.880 to 0.966) | 0.080 (0.026 to 0.262) | 4.6x10^-5^ |
| miR-29a-3p | 0.240 (0.133 to 0.563) | 0.021 (0.017 to 0.037) | 9.1x10^-5^ |
| miR-99b-5p | 0.636 (0.476 to 0.725) | 0.164 (0.015 to 0.359) | 1.8x10^-4^ |
| miR-125b-5p | 0.981 (0.971 to 0.988) | 0.912 (0.900 to 0.947) | 3.2x10^-4^ |
| miR-23a-3p | 0.937 (0.883 to 0.956) | 0.362 (0.215 to 0.654) | 3.2x10^-4^ |
| miR-23b-3p | 0.693 (0.581 to 0.772) | 0.223 (0.017 to 0.329) | 5.5x10^-4^ |
| miR-195-5p | 0.703 (0.344 to 0.773) | 0.025 (0.019 to 0.128) | 5.5x10^-4^ |
| miR-223-3p | 0.715 (0.560 to 0.885) | 0.054 (0.020 to 0.303) | 5.5x10^-4^ |
| miR-15a-5p | 0.855 (0.669 - 0.891) | 0.115 (0.019 - 0.337) | 1.4x10^-3^ |
| miR-29c-3p | 0.640 (0.563 - 0.705) | 0.021 (0.015 - 0.051) | 1.4x10^-3^ |
| let-7b-5p | 0.935 (0.920 - 0.961) | 0.831 (0.539 - 0.894) | 2.1x10^-3^ |
| miR-27a-3p | 0.887 (0.857 - 0.909) | 0.179 (0.059 - 0.280) | 2.1x10^-3^ |
| miR-424-5p | 0.781 (0.710 - 0.843) | 0.044 (0.015 - 0.177) | 2.1x10^-3^ |
| miR-320b | 0.925 (0.863 - 0.930) | 0.480 (0.400 - 0.643) | 3.1x10^-3^ |
| miR-16-5p | 0.851 (0.590 - 0.897) | 0.025 (0.019 - 0.138) | 3.1x10^-3^ |
| miR-143-3p | 0.837 (0.804 - 0.911) | 0.025 (0.019 - 0.128) | 3.1x10^-3^ |
| miR-27b-3p | 0.824 (0.760 - 0.896) | 0.217 (0.026 - 0.430) | 4.4x10^-3^ |
| miR-92a-3p | 0.892 (0.757 - 0.938) | 0.174 (0.021 - 0.446) | 4.4x10^-3^ |
| miR-101-3p | 0.900 (0.887 - 0.927) | 0.474 (0.306 - 0.564) | 6.2x10^-3^ |
| miR-150-5p | 0.727 (0.589 - 0.796) | 0.420 (0.322 - 0.59) | 8.5x10^-3^ |
| miR-1974 (obsolete) | 0.099 (0.013 - 0.392) | 0.450 (0.361 - 0.799) | 1.2x10^-2^ |
| miR-24-3p | 0.787 (0.615 - 0.861) | 0.295 (0.023 - 0.639) | 1.2x10^-2^ |
| miR-19b-3p | 0.856 (0.559 - 0.949) | 0.325 (0.021 - 0.522) | 1.6x10^-2^ |
| miR-99a-5p | 0.934 (0.861 - 0.949) | 0.829 (0.654 - 0.902) | 3.4x10^-2^ |
| miR-186-5p | 0.509 (0.248 - 0.699) | 0.283 (0.099 - 0.328) | 3.4x10^-2^ |
| miR-451a | 0.877 (0.342 - 0.987) | 0.024 (0.019 - 0.603) | 3.4x10^-2^ |
| miR-577 | 0.055 (0.007 - 0.270) | 0.314 (0.085 - 0.449) | 4.3x10^-2^ |
| miR-224-3p | 0.010 (0.007 - 0.287) | 0.247 (0.021 - 0.436) | 5.5x10^-2^ |
| miR-624-5p | 0.223 (0.045 - 0.695) | 0.675 (0.494 - 0.802) | 6.8x10^-2^ |
| miR-616-5p | 0.010 (0.008 - 0.228) | 0.250 (0.061 - 0.349) | 6.8x10^-2^ |
| miR-526b-5p | 0.564 (0.102 - 0.66) | 0.764 (0.397 - 0.929) | 1.0x10^-1^ |
| miR-20a-5p | 0.635 (0.163 - 0.701) | 0.413 (0.171 - 0.57) | 1.5x10^-1^ |
| miR-26a-5p | 0.727 (0.658 - 0.801) | 0.684 (0.545 - 0.737) | 2.4x10^-1^ |
| miR-662 | 0.328 (0.140 - 0.478) | 0.080 (0.025 - 0.444) | 2.4x10^-1^ |
| miR-132-3p | 0.419 (0.013 - 0.624) | 0.026 (0.015 - 0.168) | 2.7x10^-1^ |
| miR-886-5p (obsolete) | 0.179 (0.007 - 0.602) | 0.180 (0.021 - 0.734) | 2.7x10^-1^ |
| miR-1979 (obsolete) | 0.835 (0.614 - 0.897) | 0.890 (0.794 - 0.946) | 3.2x10^-1^ |
| miR-30b-5p | 0.702 (0.646 - 0.774) | 0.589 (0.375 - 0.774) | 3.2x10^-1^ |
| miR-34c-5p | 0.532 (0.063 - 0.696) | 0.237 (0.024 - 0.406) | 3.2x10^-1^ |
| miR-1912 | 0.015 (0.010 - 0.491) | 0.128 (0.025 - 0.249) | 3.6x10^-1^ |
| miR-495-3p | 0.033 (0.011 - 0.436) | 0.107 (0.023 - 0.422) | 4.6x10^-1^ |
| miR-152 | 0.545 (0.013 - 0.798) | 0.025 (0.019 - 0.162) | 4.6x10^-1^ |
| miR-1911-5p | 0.182 (0.013 - 0.434) | 0.024 (0.018 - 0.094) | 4.6x10^-1^ |
| miR-204-5p | 0.994 (0.991 – 1.000) | 1.000 (0.991 – 1.000) | 5.1x10^-1^ |
| miR-30c-5p | 0.729 (0.589 - 0.773) | 0.468 (0.386 - 0.821) | 5.1x10^-1^ |
| let-7g-5p | 0.265 (0.032 - 0.647) | 0.108 (0.021 - 0.301) | 5.1x10^-1^ |
| miR-491-5p | 0.103 (0.009 - 0.291) | 0.194 (0.026 - 0.310) | 5.1x10^-1^ |
| miRPlus-A1031 | 0.622 (0.010 - 0.936) | 0.817 (0.434 - 0.945) | 5.7x10^-1^ |
| miR-222-3p | 0.193 (0.048 - 0.583) | 0.078 (0.021 - 0.358) | 5.7x10^-1^ |
| miR-142-3p | 0.569 (0.224 - 0.777) | 0.380 (0.292 - 0.557) | 6.3x10^-1^ |
| miR-30b-3p | 0.391 (0.188 - 0.712) | 0.557 (0.226 - 0.696) | 7.0x10^-1^ |
| miR-708-5p | 0.326 (0.009 - 0.610) | 0.122 (0.020 - 0.444) | 7.0x10^-1^ |
| miR-26b-5p | 0.092 (0.013 - 0.552) | 0.036 (0.020 - 0.204) | 7.0x10^-1^ |
| miR-140-3p | 0.744 (0.170 - 0.838) | 0.490 (0.398 - 0.834) | 7.6x10^-1^ |
| miR-1909-3p | 0.735 (0.158 - 0.860) | 0.420 (0.026 - 0.908) | 7.6x10^-1^ |
| miR-182-5p | 0.146 (0.013 - 0.284) | 0.065 (0.019 - 0.343) | 7.6x10^-1^ |
| miR-181a-5p | 0.136 (0.008 - 0.339) | 0.099 (0.020 - 0.279) | 7.6x10^-1^ |
| miR-191-5p | 0.329 (0.057 - 0.535) | 0.349 (0.057 - 0.488) | 8.3x10^-1^ |
| miR-185-5p | 0.119 (0.010 – 0.539) | 0.030 (0.020 – 0.141) | 9.7x10^-1^ |
|  |  |  |  |
|  |  |  |  |
